# Temporal patterns of sleep latency in central hypersomnia and attention deficit hyperactivity disorder: a cluster analysis exploration using Multiple Sleep Latency Test

**DOI:** 10.3389/fpsyt.2024.1361140

**Published:** 2024-03-13

**Authors:** Takashi Maruo, Shunsuke Takagi, Sunao Uchida, Hidehiko Takahashi, Genichi Sugihara

**Affiliations:** ^1^ Department of Psychiatry and Behavioral Sciences, Tokyo Medical and Dental University Graduate School, Tokyo, Japan; ^2^ Sunao Clinic, Saitama, Japan; ^3^ Sleep Research Institute, Waseda University, Tokyo, Japan; ^4^ Faculty of Sport Sciences, Waseda University, Saitama, Japan; ^5^ Center for Brain Integration Research, Tokyo Medical and Dental University, Tokyo, Japan

**Keywords:** excessive daytime sleepiness, time series clustering, hierarchical clustering, machine learning, data-driven approach

## Abstract

**Introduction:**

Excessive daytime sleepiness (EDS) is a crucial symptom that diminishes the quality of life. The primary causes of EDS are central hypersomnia, including narcolepsy type 1 (NT1), type 2 (NT2), and idiopathic hypersomnia (IH). EDS is often associated with other psychiatric disorders, particularly attention deficit hyperactivity disorder (ADHD). The Multiple Sleep Latency Test (MSLT) is the standard assessment tool for EDS. Although the MSLT yields numerous parameters, most are not employed in clinical practice. In this study, we leveraged novel MSLT parameters to discern central hypersomnia and ADHD presence. Our analysis focused on sleep latency variability and employed cluster analysis to identify unique temporal patterns.

**Methods:**

We examined the MSLT data from 333 patients; of these, 200 (aged 14–54, mean: 24.9 ± 8.1, years; 114 females) met the inclusion criteria comprising comprehensive data an Apnea-Hypopnea Index (AHI) below 5, and no prior diagnosis of sleep apnea syndrome. We employed a time-course cluster approach that specifically targeted sleep latency variability during the MSLT.

**Results:**

Considering both multiple clustering quality evaluations and the study’s objectives, we identified 9 distinct clusters. Clusters 1 and 3 predominantly had MSLT-positive results; Cluster 2 was entirely MSLT-positive; Clusters 4, 5, 6, 8, and 9 were mainly MSLT-negative; and Cluster 7 had mixed results. The diagnosis of hypersomnia varied notably among Clusters 1, 2, 3, and 7, with Cluster 2 demonstrating a pronounced tendency towards NT1 and NT2 diagnoses (p < 0.005). However, no significant correlation was observed between ADHD diagnoses and specific sleep latency patterns in any cluster.

**Conclusions:**

Our study highlights the value of time-course clustering in understanding sleep latency patterns of patients with central hypersomnia.

## 1 Introduction

Excessive daytime sleepiness (EDS) is a crucial symptom associated with various sleep disorders, and the diagnosis of the underlying disorder is challenging (1, 2). EDS leads to reduced productivity, lower academic performance, increased risk of accidents during hazardous tasks, and subjective discomfort (3–5). The primary cause of EDS is central hypersomnia, including narcolepsy type 1 (NT1), narcolepsy type 2 (NT2) and idiopathic hypersomnia (IH). The pathophysiology of NT1 is relatively well-established and characterized by the loss of orexin-producing neurons in the hypothalamus. However, substantial knowledge gaps persist regarding the pathophysiologies of NT2 and IH (6). Given these knowledge deficiencies, the diagnosis of central hypersomnia is ambiguous for differentiating between various disorders within this category.

The objective measurement of symptoms and features is essential for the precise diagnosis and evaluation of central hypersomnia. However, existing testing methodologies are not ideal for accomplishing this task. The Multiple Sleep Latency Test (MSLT) is a widely accepted tool for assessing the symptoms and features of central hypersomnia and the diagnostic criteria are recognized for this disorder in the International Classification of Sleep Disorders 3rd Edition (ICSD-3) (7). The MSLT assesses the ease of falling asleep as a proxy for EDS intensity, quantified by sleep latency and minutes patients take to fall asleep. The MSLT consists of 4 to 5 daytime naps with the mean sleep latency (mSL) across these naps, which determines the test outcome. The mSL is employed as a diagnostic criterion for central hypersomnia with a threshold of 8 minutes. The MSLT also provides information on the occurrence of the sleep-onset rapid eye movement period (SOREMP), which is characteristic of NT1 and NT2. Furthermore, the MSLT can yield more detailed information, including absolute mSL length (8) and variations in sleep latency among individual naps. The former may reflect sleepiness strength and the latter might reflect lack of sleep or other sleep pathologies. However, these additional parameters are not currently utilized in clinical decision-making (7). These can be potential diagnostic tools for distinguishing central hypersomnia.

While the distinctions between NT1, NT2, and IH remain pivotal, the intricate relationship between EDS and psychiatric disorders, particularly attention deficit hyperactivity disorder (ADHD), adds another layer of complexity (9). Recent studies have shown a strong coexistence between EDS and ADHD (10–12). For example, 61% of the patients with central hypersomnia with EDS exhibit ADHD-like symptoms (13), whereas 38% of individuals with ADHD have EDS (10). The coexistence of ADHD and EDS remains challenging to diagnose with an objective sleep examination. Although some studies found no significant differences in mSL (14, 15), others have reported shorter mSL in patients with ADHD (16, 17). One study reported increased variance in sleep latency for each MSLT nap of ADHD individuals (14). These studies suggest that additional parameters determined by the MSLT can be useful for assessing the coexistence of ADHD.

In this study, we derived a novel approach to distinguish central hypersomnia and ascertain the presence of ADHD implementing formerly unused parameters from the MSLT. We specifically analyzed temporal patterns of sleep latency (in seconds) during the MSLT as additional parameters, employing hierarchical clustering based on Euclidean distances to group data points with similar sleep latency patterns into distinct groups. Its efficacy in elucidating underlying mechanisms and facilitating targeted treatment strategies has been reported (18–22). We applied a cluster analysis to the sleep latency temporal patterns of the MSLT from individuals with EDS, both with and without ADHD. Exploring distinct MSLT subgroups based on a cluster analysis may enhance our understanding of the interplay between EDS and ADHD and provide deeper insights into central hypersomnia.

## 2 Materials and methods

This study was conducted at the Sunao Clinic, Saitama, Japan, followed the Declaration of Helsinki, and was approved by the ethics committee of Tokyo Medical and Dental University. As this was a retrospective study, the committee waived the need for informed consent from the participants.

### 2.1 Participants

We collected data from consecutive patients with EDS who underwent MSLT at Sunao Clinic between January 2020 and December 2021. The MSLT was employed based on the clinical need for patients complaining of daytime sleepiness and all included MSLTs were for initial diagnosis; patients undergoing repeat MSLT examinations were not included. Data were extracted from their medical records. Patients with incomplete MSLT data were excluded. In addition, patients with an Apnea-Hypopnea Index (AHI) of 5 or higher or a patient with a previous diagnosis of sleep apnea were excluded due to the possibility of prolonged sleep latency on the MSLT of patients with sleep apnea syndrome (23).

### 2.2 Clinical information

Physicians specializing in psychiatry-based sleep conducted medical interviews to diagnose sleep and psychiatric disorders. The obtained medical history included information on sleep habits, related complaints, types of sleep problems, and history of symptoms related to ADHD. In addition, the order of daytime sleepiness onset and ADHD-like symptoms was obtained to determine whether inattention symptoms were present before the emergence of daytime sleepiness. The following information was collected at the time of assessment: age, sex, height, weight, body mass index (BMI), presence of comorbid psychiatric disorders, Epworth Sleepiness Scale (ESS) score, and medications, if any. Patients who were primarily diagnosed with mood or anxiety disorders by psychiatrists were excluded from the MSLT testing and consequently from the study.

### 2.3 MSLT

The MSLT was conducted after least 6 hours of sleep and 7 or more hours spent in bed on the prior night. The total sleep time was confirmed by objective night sleep monitoring using polysomnography. AHI was determined using polysomnography. The MSLT nap tests were conducted following the standard protocol recommended by the American Academy of Sleep Medicine (AASM) (24). MSLT nap tests were conducted using an Alice 6 LDx (Koninklijke Phillips, Amsterdam, Netherlands) system. A total of 4 or 5 naps were performed at 2-hour intervals, and sleep latency and occurrence of SOREMPs were recorded. Patients taking medications that could affect their sleep were encouraged to discontinue or reduce their medications as much as possible before the test. No patients were taking ADHD medication at the time of the MSLT test. The sleep latency of the first four naps was used for the analysis.

### 2.4 Diagnosis of central hypersomnia and ADHD

Physicians specializing in psychiatry-based sleep medicine diagnosed central hypersomnia according to ICSD-3 (7). The diagnosis of central hypersomnia included NT1, NT2, and IH, as determined by the ICSD-3. ADHD diagnosis was conducted at an initial clinic visit using the Diagnostic and Statistical Manual of Mental Disorders, Fifth Edition (DSM-5) criteria (25), involving direct clinical interviews by psychiatrists. ADHD subtypes classification was also made at an initial clinic visit. No structured interviews were used.

### 2.5 Cluster analysis

We conducted a cluster analysis of the temporal patterns of sleep latency of each session of the MSLT and generated a dendrogram using R software and the RStudio platform (26). Specifically, hierarchical clustering was adopted (27) and Euclidean distances were calculated.

Two methods were employed to determine the number of clusters. Method 1 involved four metrics to assess clustering quality; [1] The silhouette method was utilized to assess both the closeness of data points within a cluster and their separation from other clusters. [2] The elbow method was used to determine the point at which the within-cluster variance no longer decreases significantly with the addition of another cluster — suggesting a natural division within the data. [3] The gap statistic compared the total intra-cluster variance against expected values under a null hypothesis, identifying a suitable number of clusters where a significant ‘gap’ occurs. [4] The Dunn Index was applied to identify clusters that are well-separated and compact, by examining the ratio between the minimal inter-cluster distances to maximal intra-cluster distances. Each of these metrics provides a different perspective on the clustering structure, and together they offer a comprehensive view to guide the selection of the number of clusters (18, 28, 29). In Method 2, we chose the number of clusters which made sense from a clinical perspective, particularly in relation to distinct patterns in MSLT sleep latency and trends in sleep medicine and ADHD diagnoses, because relying solely on statistical metrics in Method 1 might not fully capture the specific clinical context of our study. The primary aim of this investigation was to assess the presence of characteristic patterns in time-series changes in MSLT sleep latency and to examine any trends in sleep medicine and ADHD diagnoses associated with each pattern. To strike a balance between the evaluation metrics and the study objectives, the final number of clusters was chosen considering both statistical validation (Method 1) and clinical relevance (Method 2).

### 2.6 Statistical analyses

Chi-square tests were utilized to evaluate differences in the presence and type of central hypersomnia between ADHD and typical development, as well as the distribution of hypersomnia and ADHD within each cluster. Patient characteristics, including age, AHI, ESS scores, and BMI, were first assessed for normality and homogeneity of variance using the Kolmogorov-Smirnov and Shapiro-Wilk tests. Depending on the results, a 1-way analysis of variance or Kruskal-Wallis test was applied to examine the significant differences among clusters for these parameters. In cases where significant differences were found, *post hoc* analyses were conducted. The sex ratio across clusters was assessed using a chi-square test. All statistical analyses were performed using IBM SPSS Statistics for Windows, Version 27.0 (IBM Corp., Armonk, NY, USA), with p-values < 0.05 denoting statistical significance.

## 3 Results

**Figure 1:**
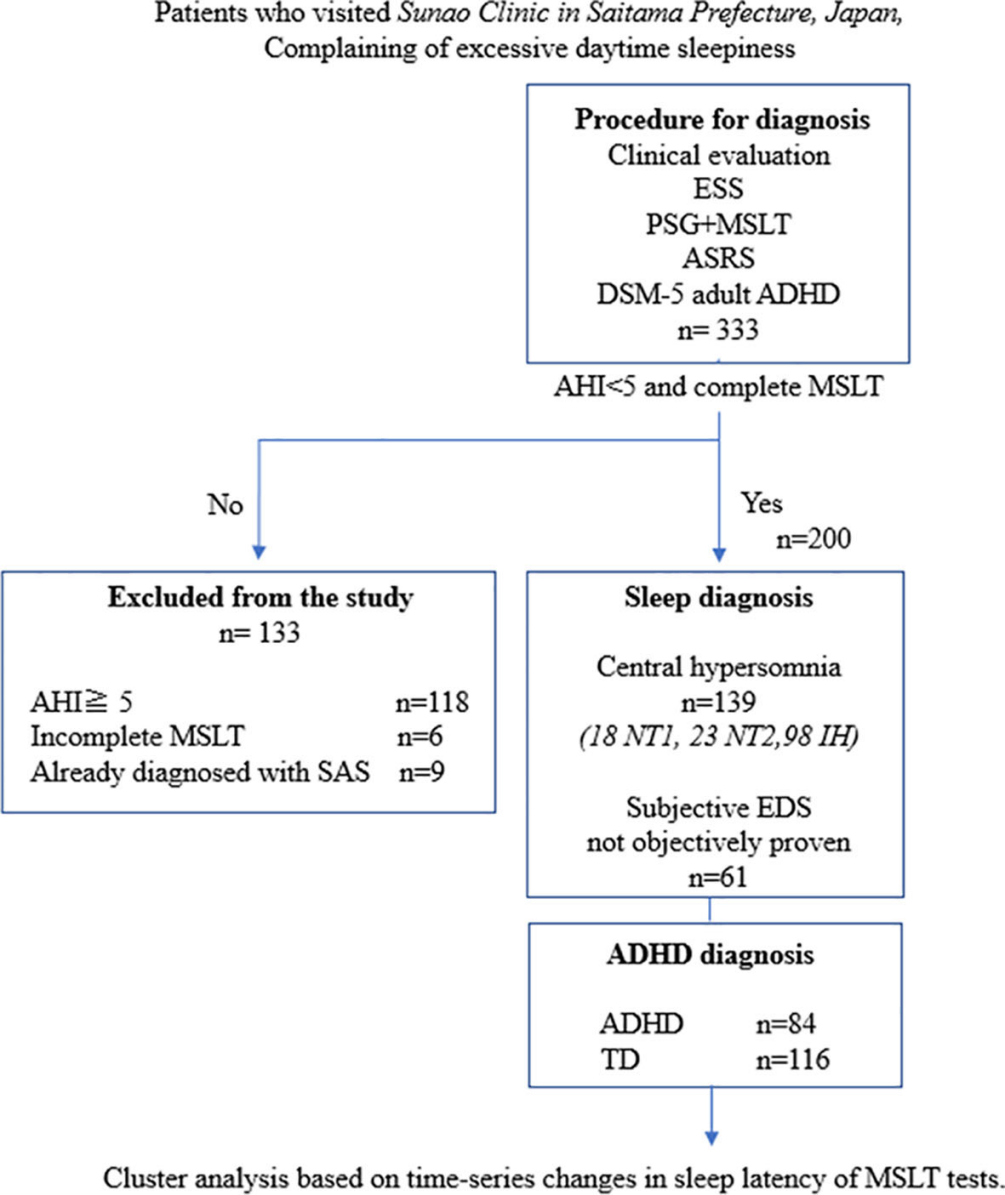
Study flowchart. ADHD, attention deficit hyperactivity disorder; ASRS, Adult ADHD Self-Report Scale Version 1.1; DSM-5, Diagnostic and Statistical Manual of Mental Disorders, Fifth Edition; ESS, Epworth Sleepiness Scale; IH, idiopathic hypersomnia; MSLT, Multiple Sleep Latency Test; NT1, narcolepsy type 1; NT2, narcolepsy type 2; PSG, polysomnography; SAS, sleep apnea syndrome; TD, typical development.

**Figure 2:**
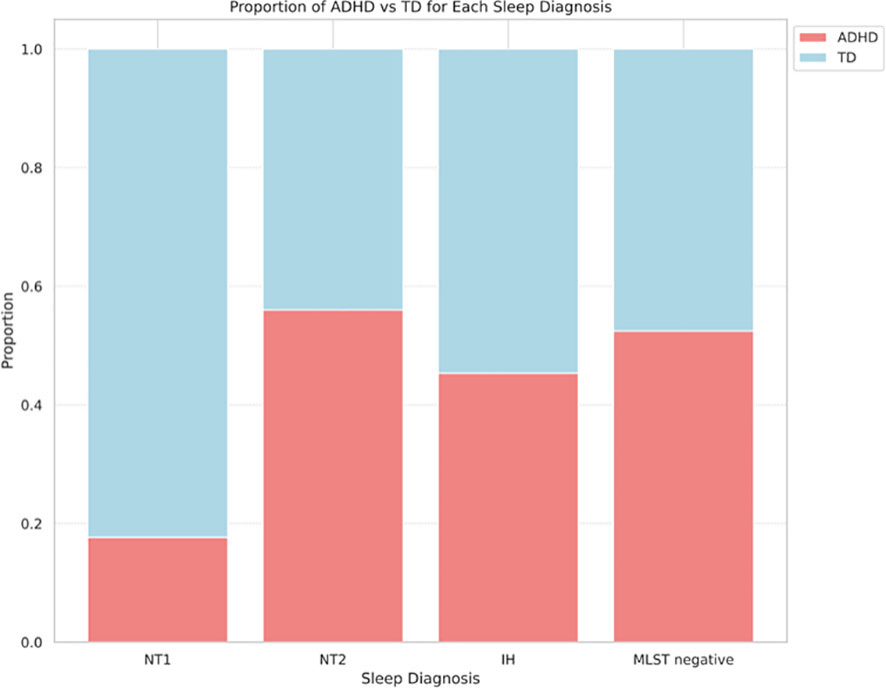
Comparison of ADHD and TD ratios for each hypersomnia diagnosis (P=0.057). ADHD, attention deficit hyperactivity disorder; IH, idiopathic hypersomnia; MSLT, Multiple Sleep Latency Test; NT1, narcolepsy type 1; NT2, narcolepsy type 2; TD, typical development.

During the study period, 333 patients underwent MSLT, with 200 patients included in the analysis after applying exclusion criteria (**Figure 1**). **Table 1** presents the demographic characteristics of the participants. Their age range was 14–54 (mean ± SD; 24.9 ± 8.1) years; 114 female and 86 male patients. Among them, 139, 17, 25, and 97 were diagnosed with central hypersomnia, NT1, NT2, and IH, respectively. The remaining 61 patients did not meet the hypersomnia diagnosis criteria. There were no cases in our study suspected of long sleep duration type IH, which would require a 48-hour polysomnography for diagnosis. In terms of ADHD diagnosis, 93 patients were diagnosed as having the disorder, while 107 were categorized as typically developing. The ADHD diagnoses were further subdivided into inattentive type (ADHD-I):78, combined type (ADHD-C):14, and hyperactive-impulsive type (ADHD-HI):1. In each sleep disorder category, the proportion of typically developing versus ADHD-diagnosed individuals—NT1 (82.4% vs. 17.7%), NT2 (44.0% vs. 56.0%), IH (54.6% vs. 45.4%), and negative hypersomnia (47.5% vs. 52.5%)—exhibited marginal differences that did not reach statistical significance (p = 0.057), as illustrated in **Figure 2**.

**Table 1:** Summary of demographic and clinical variables.

Variable	N	%
Age (years)		
14–19 20–29 30–39 40+	68 93 28 11	34 46.5 14 5.5
Sex		
Males Females	86 114	43 57
BMI		
-18.5 18.5–25 25+ n/a	37 130 30 3	18.5 65 15 1.5
mSL		
-5 min 5–8 min 8+ min	84 55 61	42 27.5 30.5
Number of SOREMP		
0 1 2 3 4	136 22 20 13 9	68 11 10 6.5 4.5
Sleep diagnosis		
NT1 NT2 IH Negative	17 25 97 61	8.5 12.5 48.5 30.5
Developmental disorder diagnosis		
ADHD ADHD-I ADHD-C ADHD-HI Typical development	93 78 14 1 107	46.5 53.5
ESS		
-9 10- n/a	47 152 1	23.5 76 0.5
ASRS		
A -3 4- n/a	117 81 2	58.5 40.5 1.1

ADHD, attention deficit hyperactivity disorder; ADHD-I, ADHD inattentive type; ADHD-C, ADHD combined type; ADHD-HI, hyperactive-impulsive type; ASRS, Adult ADHD Self-Report Scale Version 1.1; BMI, body mass index; ESS, Epworth Sleepiness Scale; mSL, mean sleep latency; IH, idiopathic hypersomnia; MSLT, Multiple Sleep Latency Test; NT1, narcolepsy type 1; NT2, narcolepsy type 2; SOREM, sleep-onset rapid eye movement period.

In determining the optimal number of clusters for our study, we initially employed Method 1 described above. These measures suggested a range of optimal clusters from 2 to 10; the silhouette method suggested 2 clusters; the elbow method 8 or 9; the gap statistics 2, 9, and 10; and the Dunn Index indicated 7 as the most cohesive and separated solution. However, these did not provide a clear conclusion on the best number. Therefore, we complemented this approach with Method 2. Starting with a single cluster, we incrementally increased the number of clusters, assessing the data at each step. We selected a nine-cluster solution, as it provided a detailed and clinically meaningful differentiation of the sleep latency patterns without overcomplicating the structure. The dendrogram created by the clustering process is described in **Supplementary Figure 1**.

Demographic data, MSLT outcomes, and ADHD diagnosis distribution across the clusters are detailed in **Table 2**. There were no statistically significant differences in the distribution of typical development and ADHD, age, AHI, BMI, ESS, or sex distribution between clusters. Clusters 1 and 3 predominantly consisted of MSLT-positive cases (mSL < 8 minutes), while Cluster 2 was entirely MSLT-positive. In contrast, Clusters 4, 5, 6, 8, and 9 demonstrated MSLT-negative results (mSL > 8 minutes). Cluster 7 exhibited an even distribution between MSLT-positive and MSLT-negative cases.

**Table 2:** Sleep diagnosis and presence of ADHD for each cluster.

	*Cluster 1*	*Cluster 2*	*Cluster 3*	*Cluster 4*	*Cluster 5*	*Cluster 6*	*Cluster 7*	*Cluster 8*	*Cluster 9*
*Number of Participants*	17	60	70	11	10	14	4	6	8
*Age (years)* *(Average ± SD)*	22.2 ± 5.1	26.2 ± 8.6	25.5 ± 8.2	20.9 ± 5.4	26.7 ± 10.2	24.0 ± 9.6	24.1 ± 0.7	22.0± 7.2	23.3 ± 7.1
*Sex (M/F, %)*	13/4 76.5%/23.5%	25/35 41.7%/58.3%	30/40 42.9%/57.1%	4/7 36.4%/63.6%	4/6 40.0%/60.0%	4/10 28.6%/71.4%	0/4 0.0%/100.0%	4/2 66.7%/33.3%	2/6 25.0%/75.0%
*MSLT mSL(sec)* *(Average ± SD)*	375.6 ± 96.6	134.7± 62.2	365.7 ± 94.1	734.0 ± 102.6	631.2 ± 90.6	752.8 ± 119.1	514.2 ± 97.0	675.5 ± 67.8	1057.2 ± 106.4
*Sleep Diagnosis (n, %)*	IH:15 (88.2%) negative:2 (11.8%)	NT1:16 (26.7%) NT2:20 (33.3%) IH: 24 (40.0%)	NT1:1 (1.4%) NT2:5 (7.1%) IH:56 (80.0%) negative:8 (11.4%)	negative:11 (100%)	negative:10 (100%)	negative:14 (100%)	IH:2 (50.0%) negative:2 (50.0%)	negative:6 (100%)	negative:8 (100%)
*ADHD Diagnosis (n, %)*	ADHD:10 (58.8%) TD:7 (41.2%)	ADHD:24 (40.0%) TD:36 (60.0%)	ADHD:34 (48.6%) TD:36 (51.4%)	ADHD:6 (54.5%) TD:5 (45.5%)	ADHD:2 (20.0%) TD:8 (80.0%)	ADHD:6 (42.9%) TD:8 (57.1%)	ADHD:3 (75.0%) TD:1 (25.0%)	ADHD:5 (83.3%) TD:1 (16.7%)	ADHD:3 (37.5%) TD:5 (62.5%)
*ESS (Average ± SD)*	11.7 ± 3.8	13.7 ± 4.5	12.0 ± 4.7	9.3 ± 4.6	10.3 ± 3.2	13.0 ± 4.8	10.8 ± 1.0	10.5 ± 3.4	12.6 ± 4.1
*BMI (Average ± SD)*	20.2 ± 1.8	22.6 ± 3.5	21.2 ± 3.9	20.0 ± 2.0	21.7 ± 3.3	21.0 ± 3.0	21.0 ± 0.9	20.1 ± 2.1	20.9 ± 3.4

ADHD, attention deficit hyperactivity disorder; BMI, body mass index; ESS, Epworth Sleepiness Scale; IH, idiopathic hypersomnia; mSL, mean sleep latency; MSLT, Multiple Sleep Latency Test; NT1, narcolepsy type 1; NT2, narcolepsy type 2; TD, typical development.

**Figure 3:**
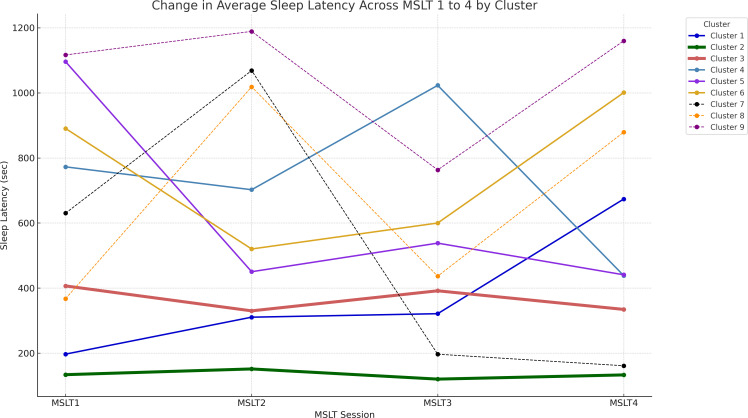
Illustration of the fluctuation in sleep latency during each MSLT session. Average sleep latency values are plotted for patients in clusters 1 through 9. Line thickness represents the number of participants in each cluster. MSLT, Multiple Sleep Latency Test.

**Figure 4:**
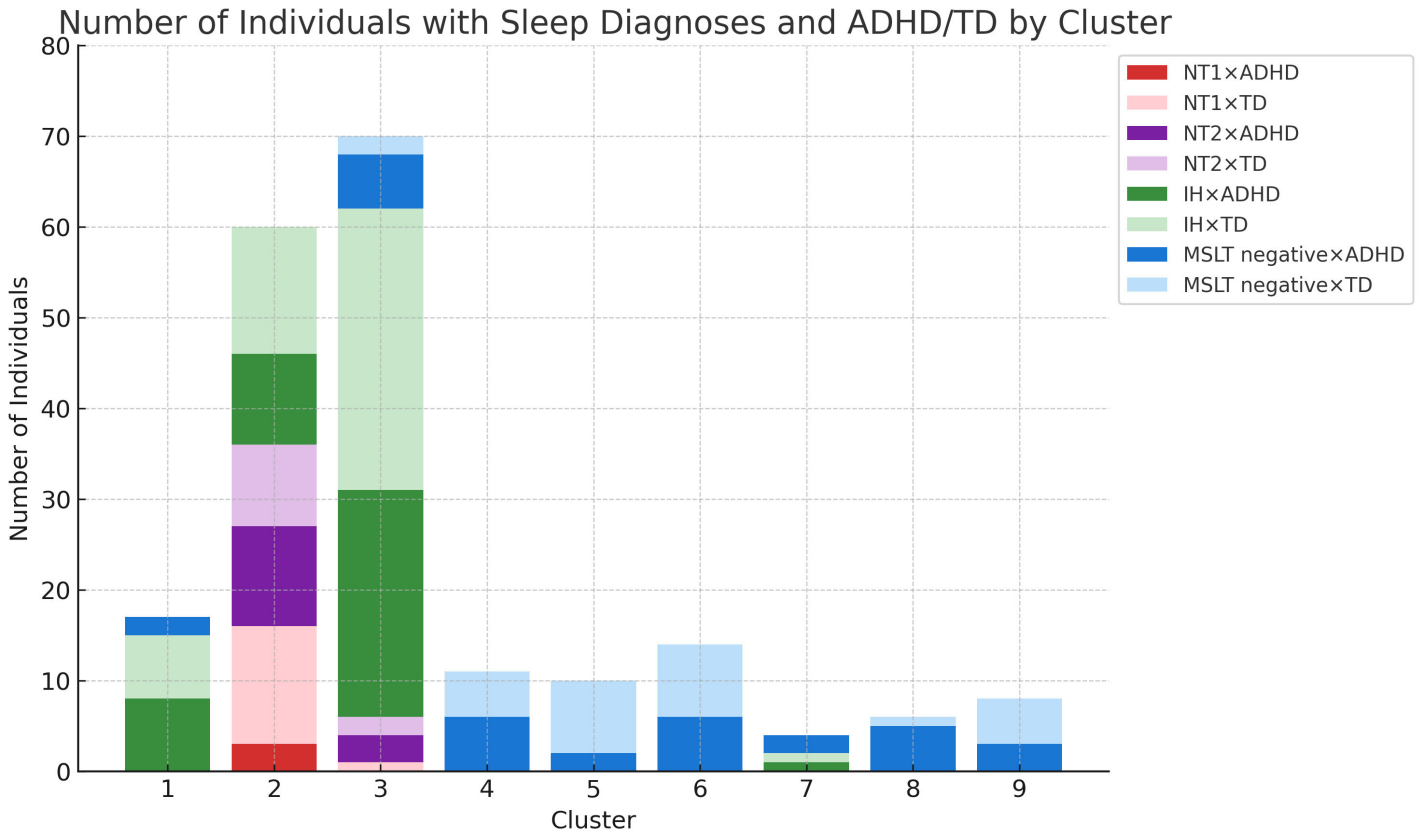
Sleep disorders and ADHD combinations per cluster. Number of Individuals with Sleep Diagnosis and ADHD/TD by Cluster - This graph presents the actual count of individuals categorized into each sleep disorder (IH, NT1, NT2) and ADHD/TD across different clusters. ADHD, attention deficit hyperactivity disorder; IH, idiopathic hypersomnia; NT1, narcolepsy type 1; NT2, narcolepsy type 2; TD, typical development.

Patterns of sleep latency fluctuations across the clusters are illustrated in **Figure 3**. Cluster 1 included 17 participants, 15 of whom were diagnosed with IH; sleep latency increased from the first to the fourth session. Cluster 2 had 60 participants; 16 with NT1, 20 with NT2, and 24 with IH; sleep latency remained consistently short across sessions. Cluster 3 consisted of 70 participants, with 62 patients diagnosed with hypersomnia; 56 with IH, 1 with NT1, 5 with NT2; sleep latencies were consistently longer than those in Cluster 2 but remained within 8 minutes. Cluster 4 included 11 participants with consistently long sleep latencies, except for a slight shortening in the fourth session. Cluster 5 had 10 participants, starting with a very long sleep latency in the first session and stabilizing at approximately 8–9 minutes from the second session onward. Cluster 6 had 14 participants, with sleep latencies consistently longer than 8 minutes, especially long in the first and fourth sessions. Cluster 8 and 9 consisted of a small number of participants. Cluster 7 included 4 participants, with half diagnosed with IH. Their sleep latency notably shortened during the afternoon sessions, specifically the third and fourth. **Figure 4** and **Supplementary Figure 2** illustrate the distribution of sleep and ADHD diagnoses across Clusters 1 through 9.

We further investigated the clinical characteristics of the clusters. First, we focused on Clusters 1, 2, and 3. Again, the tests revealed no significant differences for the proportion of participants with typical development and those with ADHD (p = 0.337). Next, we evaluated whether there was a significant difference in hypersomnia diagnoses based on sleep latency patterns among Clusters 1, 2, and 3. The results showed a statistically significant difference in diagnoses among these clusters (p < 0.005). *Post hoc* analysis showed that Cluster 2 had a pronounced inclination towards NT1 and NT2 diagnoses (p < 0.005). In contrast, Cluster 1, which did not display a dominant trend similar to Cluster 2, exhibited a unique distribution pattern, especially considering the lack of NT1 and NT2 diagnoses.

## 4 Discussion

This study differentiated between central hypersomnia and the coexistence of ADHD by analyzing the temporal patterns in sleep latency during the MSLT. Using hierarchical clustering, we explored sleep latency patterns in patients with EDS and investigated the relationship between these patterns and the diagnoses of central hypersomnia and ADHD. Our analysis revealed 9 distinct clusters, with patients with central hypersomnia primarily classified within Clusters 1, 2, and 3, while Clusters 4–9 predominantly included participants without a hypersomnia diagnosis. In contrast, clustering did not reveal a significant relationship between sleep latency patterns and ADHD diagnosis.

The features of the characteristic clusters, Clusters 1, 2, 3 and 7, are following: Cluster 1 had a unique pattern of progressively increasing sleep latency primarily among patients with IH, excluding those with narcolepsy. Unlike Clusters 2 and 3, the increased sleep latency in Cluster 1 could have reflected an adaptive response to repeated nap attempts, indicating a preserved sleep-wake regulation mechanism and a lack of specific pathophysiological features associated with narcolepsy in these individuals. In Cluster 2, their sleep latency during the MSLT exhibited a consistently short trend; this cluster included nearly all NT1 cases and the majority of NT2 cases. This observation aligns with previous research (8, 30), underscoring the utility of the MSLT as a diagnostic tool for narcolepsy. Cluster 3 included individuals with IH whose sleep latency pattern was slightly longer than that of Cluster 2. Cluster 7 also exhibited a unique pattern; long sleep latencies in morning sessions and quick sleep onset in afternoon sessions. Notably, this cluster did not include narcolepsy cases and was predominantly included those diagnosed with ADHD (3 of 4). The unique sleep latency pattern in Cluster 7 could have been caused by ADHD-related arousal instability (31, 32). Alternatively, participants in Cluster 7 had distinct circadian rhythms patterns.

Our cluster analysis did not clearly distinguish between IH and NT2. This is in line with a previous study that reported lower reproducibility for the diagnosis of NT2 and IH than that for NT1 (33). Our clustering analysis revealed that IH patients exhibit a wider range of sleep latency patterns than NT patients, such as significant fluctuations in Clusters 1 and 7, as well as more stable patterns similar to those in Clusters 2 and 3 where NT patients were predominantly found. These variations suggest the existence of distinct IH subgroups. These subgroups may be linked to underlying etiologies, prognoses, or comorbidities that align more closely with narcolepsy, warranting further investigation for a more comprehensive understanding. Our study observed a higher proportion of IH cases, while previous research suggests that IH is less common compared to narcolepsy (34). This discrepancy could be attributed to specific aspects of our research setting. Because our study was conducted at a sleep specialty clinic with a focus on psychiatry, we included a significant number of patients with psychiatric condition, including ADHD. This unique patient demographic might have contributed to the higher incidence of IH observed in our study.

Regarding ADHD, the majority of patients with ADHD who reported daytime sleepiness as their primary concern received a diagnosis of ADHD-I. This result is consistent with previous research showing that individuals with ADHD-I tend to experience higher levels of subjective sleepiness (35). Furthermore, it is important to consider the age-related changes in ADHD subtypes, as ADHD-HI decreases with age (36). In our study, the patient demographic predominantly consisted of adults, which may explain the lower occurrence of ADHD-HI in our sample. Therefore, the characteristics of our study population, including the age range and the primary symptom of daytime sleepiness, could have influenced the observed ADHD subtype distribution. In this study, no significant differences were observed in clustering between individuals with ADHD and those typically developed, which is not in line with prior literature indicating elevated subjective sleepiness in individuals with ADHD (9). This discrepancy might be attributed to the characteristics of our study population. In our outpatient sample, all reported subjective sleepiness, which may have masked the effect of ADHD on sleep latency. Compared to the ADHD prevalence of 2%-5% in the general population (37), our study demonstrated higher ADHD comorbidity rates in hypersomnia patients, as shown in **Figure 2**. This is consistent with previous studies reporting a higher prevalence of ADHD in patients with hypersomnia (38, 39). However, the extent to which ADHD comorbidity varies among the specific types of hypersomnia remains unclear. In this study, although not statistically significant (p=0.057), we found a trend that NT1 may have a lower rate of ADHD comorbidity compared to other types of hypersomnia. Further research is needed to clarify the relationships between individual hypersomnia disorders and ADHD.

This study has several limitations. First, the sample size was relatively small. Second, the study was conducted at a single sleep clinic, which might limit the generalizability of the results. Future research could benefit from multi-center collaborations and increased sample sizes for better diversity and to enhance the generalizability of these findings. Furthermore, our study focused solely on sleep latency differences in the MSLT. Additional factors, such as demographic information, medical history, and other clinical measures, such as ADHD severity, might provide a more comprehensive understanding and improve the differentiation between central hypersomnia and ADHD. This study also has the limitation of not considering the involvement of periodic limb movements. The relationship between periodic limb movements, especially in the context of ADHD and iron deficiency in relation to sleepiness and ADHD symptoms, remains a critical aspect for future research. Moreover, while this retrospective study offers valuable insights, we recognize the necessity of prospective research for a more definitive understanding of central hypersomnia and ADHD relationships. Future studies employing a prospective approach, with rigorous control over variables such as medication influence and standardized ADHD diagnostic methods, will be crucial. Such studies could provide clearer insights and enhance the robustness of findings in this complex area of sleep medicine research.

In conclusion, our findings suggested that analyzing sleep latency differences in MSLT may provide valuable insights for diagnosing central hypersomnia. Our approach, with unused parameters from the MSLT, could be utilized to identify potential subgroups of idiopathic hypersomnia. Unfortunately, our analysis did not distinguish between ADHD and typical development based on sleep latency patterns effectively. However, certain clusters hinted at the possible influence of ADHD characteristics. Future research with a larger ADHD cohort may deepen our understanding of sleep patterns in patients with ADHD, enhancing the diagnostic process.

## Data availability statement

The raw data supporting the conclusions of this article will be made available by the authors, without undue reservation.

## Ethics statement

The studies involving humans were approved by the ethics committee of Tokyo Medical and Dental University. The studies were conducted in accordance with the local legislation and institutional requirements. As this was a retrospective study, the committee waived the need for informed consent from the participants.

## Author contributions

TM: Conceptualization, Data curation, Formal analysis, Investigation, Methodology, Project administration, Resources, Validation, Visualization, Writing – original draft, Writing – review & editing. ST: Methodology, Supervision, Writing – original draft, Writing – review & editing. SU: Methodology, Project administration, Supervision, Writing – original draft, Writing – review & editing. HT: Funding acquisition, Supervision, Validation, Writing – original draft, Writing – review & editing. GS: Methodology, Supervision, Writing – original draft, Writing – review & editing, Conceptualization, Data curation, Investigation, Validation, Visualization.
