# Supplementary material for: Temporal patterns of sleep latency in central hypersomnia and attention deficit hyperactivity disorder: a cluster analysis exploration using Multiple Sleep Latency Test
Source: Front Psychiatry. 2024 Mar 13;15:1361140. doi: 10.3389/fpsyt.2024.1361140 (PMC10966118; doi:10.3389/fpsyt.2024.1361140)
Supplement: Supplementary file 1 [file DataSheet_1.pdf]

Supplementary Material

Temporal Patterns of Sleep Latency in Central Hypersomnia and Attention Deficit Hyperactivity Disorder: A Cluster Analysis Exploration Using Multiple Sleep Latency Test

Takashi Maruo1,2, Shunsuke Takagi1,2,3, Sunao Uchida1,2,3,4, Hidehiko Takahashi1,5, Genichi Sugihara1*

*** Correspondence:** Genichi Sugihara email: gen-psyc@tmd.ac.jp

1.1 Supplementary Figures

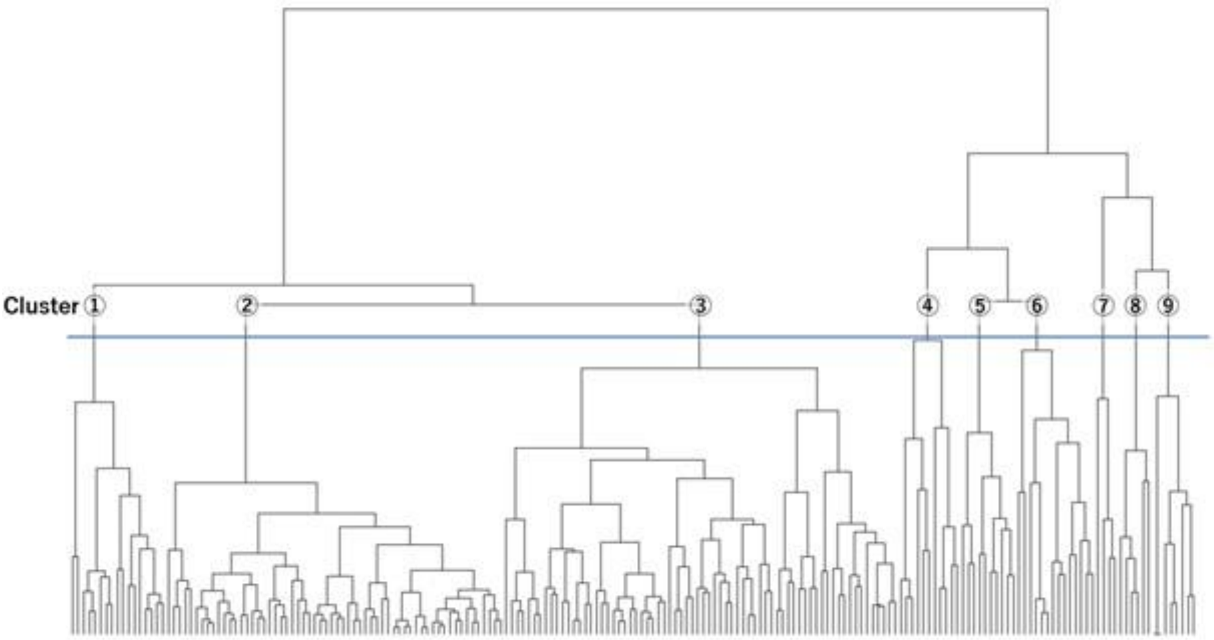

Supplementary Figure 1. Hierarchical clustering of patients into distinct clusters

This dendrogram results from the hierarchical clustering analysis, illustrating how patients were grouped into distinct clusters. The dendrogram was cut at an optimal level to form 9 clusters. The 9 clusters were numbered from the left side of the dendrogram as clusters 1 through 9.

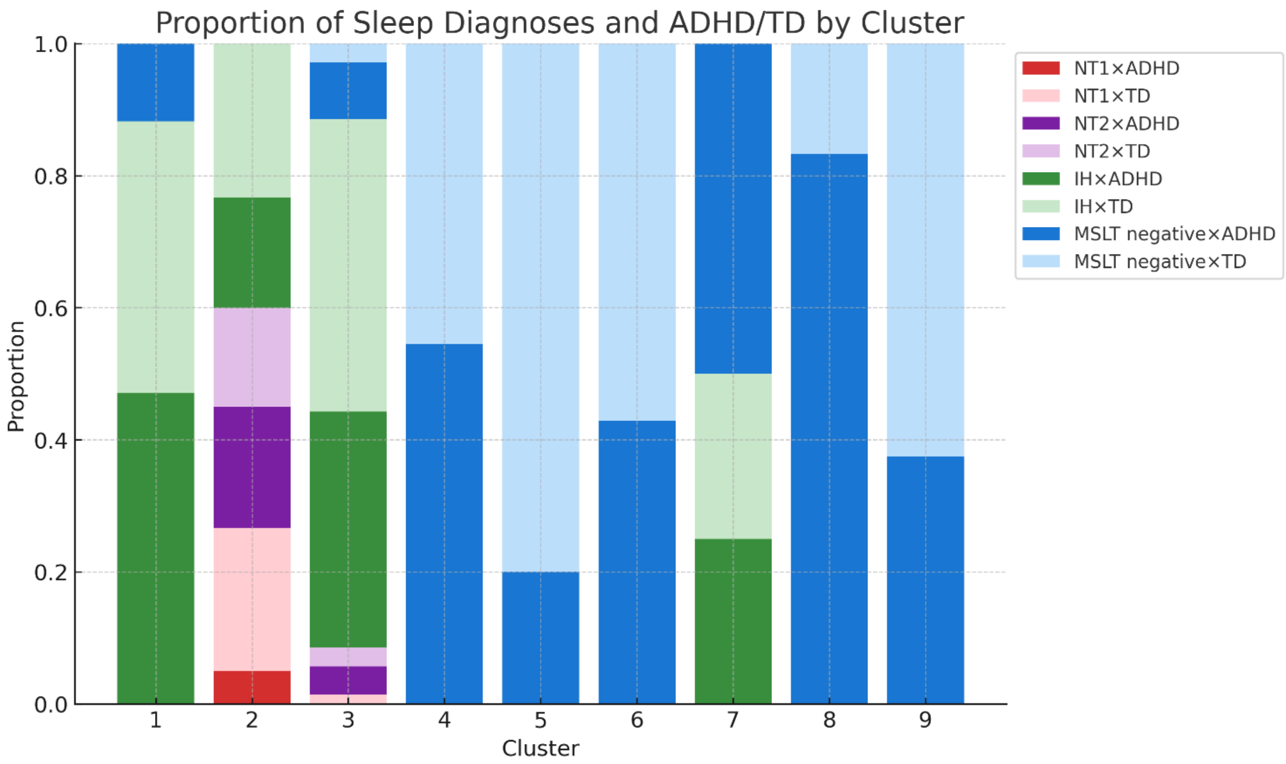

Supplementary Figure 2. Sleep disorders and ADHD combinations per cluster

Proportion of Sleep Diagnosis and ADHD/TD by Cluster - This graph shows the percentage distribution of sleep disorders (IH, NT1, NT2) and ADHD/TD across different clusters.

ADHD, attention deficit hyperactivity disorder; IH, idiopathic hypersomnia; NT1: narcolepsy type 1; NT2: narcolepsy type 2; TD, typical development.
